# Tryptophan-Serotonin-Melatonin Pathway as a Contributor to Changes in Mood and Cognitive Functions Induced by Sleep Deprivation

**DOI:** 10.3390/ijms27125209

**Published:** 2026-06-09

**Authors:** Marcin Sochal, Aleksandra Wojtera, Marta Ditmer, Agata Gabryelska, Aleksandra Tarasiuk-Zawadzka, Szymon Turkiewicz, Filip Franciszek Karuga, Jakub Fichna, Piotr Białasiewicz

**Affiliations:** 1 Department of Sleep Medicine and Metabolic Disorders, Medical University of Lodz, 92-215 Lodz, Poland; 2 Department of Biochemistry, Medical University of Lodz, 92-215 Lodz, Polandjakub.fichna@umed.lodz.pl (J.F.)

**Keywords:** cognitive functions, melatonin, mood, serotonin, sleep, sleep deprivation, tryptophan

## Abstract

Sleep deprivation (DS) is a reduction in sleep duration due to voluntary or external factors. The mechanisms underlying the psychological and cognitive consequences of DS are complex and incompletely understood; one proposed pathway involves alterations in the serotonin (5-HT) and melatonin (MLT) systems. This study aimed to assess the effects of a single night of DS on the tryptophan (TP)-5-HT-MLT system and to examine their associations with mood and cognitive performance. Eighty healthy adults underwent polysomnography (PSG) and actigraphy-monitored DS. Blood samples, mood assessments, and cognitive tests (BEHCT, TMT, Stroop) were performed before and after PSG and DS. Levels of serotonin transporter (SERT) mRNA, TP, 5-HT, and MLT were measured. Participants were classified as Responders (RE) or Non-Responders (NR) based on post-DS mood change. DS significantly decreased TP and MLT overall. In NR, 5-HT increased and MLT decreased, unlike in RE. ΔBEHCT correlated positively with ΔTP (RE), Δ5-HT (overall), and ΔMLT (overall and RE), and negatively with ΔSERT mRNA (NR). In RE, ΔSERT mRNA negatively correlated with ΔStroop performance and positively with ΔTMT. Acute DS disrupts the TP–5-HT–MLT axis, with effects differing by mood response. These changes may influence cognitive outcomes after sleep loss.

## 1. Introduction

Sleep is a complex physiological process essential for maintaining both mental and physical health [1]. Deprivation of sleep (DS) refers to a state of insufficient or disrupted sleep arising from internal or external factors [2]. Unlike insomnia, which is a chronic sleep disorder characterized by difficulty initiating or maintaining sleep despite adequate opportunity to rest, DS typically results from voluntary or externally imposed restriction of sleep [2]. It is estimated that 9–24% of the general population experience DS, with shift work being the predominant contributor [3].

Sleep disturbances are among the most common clinical features of depression, affecting over 90% of individuals with major depressive disorder [4]. Interestingly, acute DS has been shown to induce rapid antidepressant effect [5]. Meta-analytic evidence indicates that approximately 50–80% of patients with depression exhibit an initial positive response to DS [5]. However, despite these promising outcomes, the therapeutic use of DS remains limited due to its transient nature [6].

DS significantly impacts cognitive performance across multiple domains [7]. In particular, it has pronounced adverse effects on alertness and, to a lesser extent, on executive functions [8,9]. Simple attention-based tasks, which rely on a limited number of cortical regions, appear especially vulnerable to fatigue-related decline [7]. In contrast, more complex cognitive tasks, that engage broader neural networks, may show greater resilience to the effects of short-term sleep loss [7].

The mechanisms underlying the psychological consequences of DS are complex and incompletely understood. One potential mechanism involves alterations in the serotonergic and melatoninergic pathways, both of which share tryptophan (TP) as a common precursor [10]. TP is an essential amino acid that cannot be synthesized endogenously and must therefore be obtained from dietary sources such as protein-rich foods [11]. Once absorbed, TP can be metabolized into serotonin (5-hydroxytryptamine, 5-HT) or via the kynurenine pathway [10,12]. Importantly, TP availability and its metabolic conversion are strongly influenced by the gut-brain axis, where the gut microbiota and enteroendocrine signaling modulate peripheral TP utilization and 5-HT production, thereby affecting central TP supply [13]. In the central nervous system, 5-HT acts as a key neurotransmitter, while peripherally it exerts pleiotropic effects, including modulation of immune processes that may indirectly influence mood [14]. Moreover, dysregulation in serotonergic signaling, including reduced 5-HT levels, has been implicated in affective disorders [12].

Experimental evidence indicates that DS directly enhances serotonergic neurotransmission in the hippocampus of rats, increasing extracellular and serum 5-HT concentrations [15]. Similarly, in humans, 24-h DS has been shown to elevate plasma levels of both TP and 5-HT [16]. However, relatively few studies have simultaneously assessed metabolites, mood, and cognitive performance in healthy individuals after DS. Notably, Neumeister et al. showed in depressed patients that lower TP levels following DS were associated with reduced relapse of depressive symptoms after recovery sleep, pointing to the significance of the TP-5-HT axis in the antidepressant response to DS [17].

The serotonin transporter (SERT) plays a pivotal role in regulating 5-HT availability in both synaptic and peripheral compartments [18]. In the only study addressing this topic to date, 96-h DS in rats resulted in decreased SERT activity across specific brain regions, which may potentially amplify the emotional and cognitive consequences of sleep loss [19]. Given that SERT dysregulation represents a key molecular component of depression [20], investigating its expression may provide valuable insights. Moreover, evaluating SERT mRNA expression enables the exclusion of translational control factors, offering a more direct assessment of serotonergic alterations. Although 5-HT does not cross the blood-brain barrier, evidence suggests that SERT expression in peripheral blood cells may serve as a reliable biomarker reflecting central serotonergic activity [18], thereby offering a window into the neurochemical effects of DS.

The melatoninergic pathway is responsible for the synthesis of melatonin (MLT), a hormone that plays a central role in regulating the sleep-wake cycle [21]. MLT is produced in the pineal gland from 5-HT, and its secretion is tightly modulated by light exposure [21]. Research has shown that, unlike chronic circadian disruption, a single night of DS can increase MLT levels, likely reflecting enhanced synthesis due to elevated TP availability [16,22]. Beyond its role in sleep regulation, MLT has been implicated in mood regulation and cognitive performance, with physiological MLT levels found to be significantly reduced in individuals experiencing major depressive disorder or age-related cognitive decline [23,24]. Therefore, investigating MLT dynamics in the context of DS is particularly relevant, as DS simultaneously challenges both circadian and affective regulation systems.

Elucidating the interplay between serotonergic and melatoninergic systems under conditions of DS may improve our understanding of its neurophysiological consequences. Therefore, the aim of the present study was to assess changes in SERT expression and serum concentrations of 5-HT, TP, and MLT following one night of DS, and to evaluate their associations with mood and cognitive performance.

## 2. Results

A total of 80 participants were enrolled in the study, including 49 individuals classified as RE and 31 as NR. There were no significant differences between the groups with respect to demographic or baseline characteristics, including age, sex, BMI, or smoking status. Similarly, sleep parameters such as time in bed (TIB), total sleep time (TST), and sleep latency did not differ significantly between groups. See Table 1 for details.

As shown in Table 2, analysis of the entire study group revealed a significant decrease in TP concentration from the post-sleep to the post-DS measurement (44.9 [30.4–68.2] vs. 30.8 [19.9–56.9] µmol/L; *p* = 0.024). MLT concentration also showed a significant decline over the study period (457.0 [391.7–511.2] vs. 423.6 [392.7–464.2] pg/mL; *p* = 0.043). Changes in 5-HT levels and SERT expression across the full cohort did not reach statistical significance (all *p* > 0.05).

In the RE subgroup, none of the examined parameters changed significantly. In contrast, in the NR subgroup, a significant increase in 5-HT concentration was observed (63.8 [30.7–86.7] vs. 76.6 [53.8–120.3] ng/mL; *p* = 0.043), along with a significant decrease in MLT concentration (459.3 ± 73.2 vs. 423.6 ± 57.1 pg/mL; *p* = 0.024). The reduction in TP concentration in NR approached statistical significance (44.6 [33.4–64.6] vs. 31.2 [17.9–40.9] µmol/L; *p* = 0.055). The change in SERT expression in NR was also not significant (*p* = 0.088).

**Figure 1:**
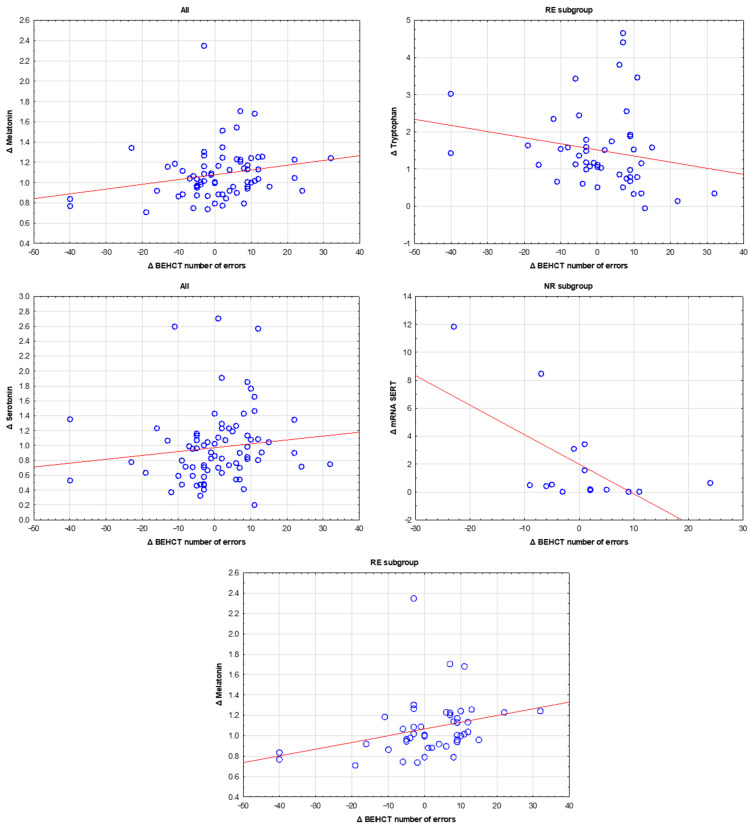
Correlation profiles of BEHCT error rates and molecular/biochemical markers between post-PSG and post-DS mornings. Abbreviations: NR—Nonresponders, RE—Responders, SERT—Serotonin Transporter, BEHCT—Bimanual Eye-hand Coordination Test, Δ—Post-DS Value/Post-PSG Value.

In the correlation analysis, most associations between changes in biochemical and molecular variables between the post-PSG and post-DS mornings and alterations in cognitive performance test scores between these two timepoints did not reach statistical significance. However, several significant correlations were identified. A positive association was observed between changes in 5-HT and MLT concentrations and BEHCT error scores in the entire study group (R = 0.25; *p* = 0.028 and R = 0.28; *p* = 0.018, respectively). Among RE participants, ΔTP was negatively correlated with ΔBEHCT errors (R = −0.32; *p* = 0.029), whereas ΔMLT demonstrated a positive correlation with ΔBEHCT errors (R = 0.41; *p* = 0.005). In the NR subgroup, ΔSERT expression was negatively correlated with ΔBEHCT errors (R = −0.54; *p* = 0.032). Figure 1 illustrates significant correlation profiles between changes in biochemical and molecular variables and BEHCT error scores between the post-PSG and post-DS mornings.

Correlations between alternations in SERT expression and performance in other cognitive tests were also observed. In the NR group, a positive association was found between changes in mRNA SERT and changes in TMT Part 1 performance (R = 0.37; *p* = 0.045). Furthermore, ΔSERT expression positively correlated with ΔTMT Part 2 performance both in the entire study population (R = 0.28; *p* = 0.014) and among RE (R = 0.38; *p* = 0.007). Additionally, in the RE subgroup, ΔSERT expression was negatively correlated with ΔStroop test Part 2 performance (R = −0.29; *p* = 0.039). For an overview, see Table 3.

## 3. Discussion

In this study, we demonstrated that even a single night of DS produced measurable alterations in peripheral TP-5-HT-MLT axis in young, healthy adults. The most prominent findings were significant decreases in TP and MLT serum concentrations in the whole group the morning after DS compared with the one post-PSG, with the reduction in MLT being particularly pronounced in the NR group. 5-HT levels and leukocyte SERT expression did not change significantly in the entire cohort, although in the NR subgroup, 5-HT levels increased and SERT expression showed a downward trend.

The observed reduction in TP concentration after one night of DS contrasts with the available literature, where DS was reported to increase circulating TP. For instance, in a rat model of acute DS in pregnant females, plasma TP levels were elevated following DS when compared with the respective control groups [25]. Similarly, human studies have revealed that extended DS under controlled laboratory conditions significantly elevated TP and 5-HT plasma concentrations [16]. Moreover, the authors interpreted these changes as a possible explanation for the rapid antidepressant effect of wake therapy [16]. That study, however, involved a small, homogeneous sample of 15 healthy men, with tightly controlled diet and lighting [16], whereas the present protocol allowed participants freedom of activity during DS. Thus, our study represents a more realistic, everyday conditions, which could account for divergent results. It is worth noting that the type of blood matrix can also influence measured TP levels, as serum generally shows higher TP concentrations than plasma [26], which may contribute to discrepancies between studies. Another plausible explanation of TP reduction in the current study may be that part of the TP reserve was rapidly utilized via alternative metabolic routes, such as the kynurenine pathway or increased 5-HT synthesis, resulting in transient decreases in circulating TP. Indeed, nocturnal wakefulness and sleep disturbances have been associated with increased activity of indoleamine 2,3-dioxygenase (IDO), which converts TP to kynurenine, leading to an elevated kynurenine/TP ratio in plasma [27]. However, this interpretation remains speculative in the absence of direct measurements of kynurenine, kynurenic acid or IDO activity in this study.

Unlike TP, 5-HT levels in the current study showed subgroup-specific alternations: median 5-HT remained unchanged overall and in the RE subgroup, whereas the NR subgroup exhibited a significant increase. These results are partially consistent with prior evidence that DS modulates serotonergic transmission. In animal models, DS has been shown to enhance 5-HT release in specific brain regions. For example, López-Rodríguez et al. demonstrated a marked increase in extracellular 5-HT levels within the rat hippocampus during 24 h of DS, which persisted into recovery sleep [28]. In humans, elevated plasma 5-HT has likewise been observed following 24 h of wakefulness [16]. Although 5-HT itself does not readily cross the blood-brain barrier, peripheral 5-HT can still influence central nervous system function indirectly: it modulates immune and inflammatory signaling, thereby altering cytokine profiles that affect brain activity, and shifting TP metabolism toward kynurenine pathways, with consequences for central 5-HT synthesis [29]. In our cohort, the isolated 5-HT increase in NR participants could reflect dysregulated or compensatory peripheral serotonergic signaling that fails to translate into clinical improvement. In contrast, RE participants maintained stable 5-HT levels, suggesting a more efficient homeostatic adaptation of the serotonergic system following DS. Future studies should integrate simultaneous measurements of peripheral and central serotonergic markers, together with kynurenine pathway analyses, to determine whether changes in peripheral 5-HT contribute to the rapid antidepressant effects of DS.

What is more, DS resulted in a significant reduction in MLT levels in the entire group. This finding is consistent with the physiology of the circadian system—MLT secretion normally occurs during darkness and nighttime wakefulness, while light exposure suppresses its release [21]. Participants in our study likely experienced environmental light stimuli during DS, what probably contributed to lower morning MLT levels relative to post-sleep measurements. In contrast, previous studies have reported increased MLT concentrations following acute DS under strictly controlled conditions, suggesting that the direction of MLT change may depend on experimental design [16]. Subgroup analysis revealed that decline in MLT levels was significant only in NR, whereas RE showed no significant change. The selective reduction in NR may reflect differences in circadian sensitivity, with these participants being more susceptible to light-induced inhibition of the suprachiasmatic nucleus-pineal pathway, leading to suppressed MLT secretion and potential desynchronization of circadian rhythms. In contrast, the stability of MLT in RE suggests more robust homeostatic and circadian regulation, which may facilitate the mood-enhancing effects of sleep loss. DS has previously been shown to disrupt the expression of core clock genes, including PER1, BMAL1, and CLOCK, which play a key role in maintaining circadian rhythmicity and regulating MLT synthesis in the pineal gland [30,31] Altered transcription of these genes may therefore contribute to the dysregulation of MLT observed after DS [30]. Moreover, changes in the expression of circadian genes have been linked to DS-linked mood regulation [31]. These findings suggest that individual differences in circadian regulation and sensitivity to light may be one of the factors that can modulate the mood effects of DS.

Marked subgroup differences, between RE and NR, were observed in both the magnitude and direction of biochemical alterations following DS. These findings suggest that mood improvement after DS is likely related to the variability in the studied molecular markers, and that the TP-5-HT-MLT pathway may underlie divergent affective responses to DS or, conversely, that mood changes themselves may exert a regulatory influence on this pathway. In this context, it is important to note that the MADRS scale, used to classify patients to subgroups, is not linearly calibrated and may therefore lack sensitivity and specificity for capturing gradual affective changes [32]. Accordingly, future studies should employ longitudinal designs with multiple post-DS assessment points and include experimental manipulations, such as supplementation with the precursor TP, to clarify temporal and causal relationships between molecular dynamics and mood after sleep loss.

While DS can transiently improve mood, it consistently impairs cognitive performance [2]. Prior studies have shown pronounced deficits in vigilance, working memory, and reaction time after even partial sleep loss [33]. Our previous study further confirmed that DS leads to marked deterioration across multiple cognitive domains, with clear subgroup differences: RE participants maintained relatively preserved cognitive efficiency, whereas NR individuals exhibited more pronounced impairments in attention and psychomotor performance [2]. Monotonous tasks relying on sustained attention appear most sensitive to DS, whereas integrative or creative tasks may be partially preserved by compensatory mechanisms [34]. In the present study, we aimed to examine whether these cognitive alterations are associated with changes in the TP-5-HT-MLT pathway. Although our cognitive test battery did not comprehensively cover all cognitive domains, the observed patterns provide valuable insight into DS-related cognitive dynamics. Increased numbers of errors in BEHCT after DS in comparison to after PSG were associated with rises in both 5-HT and MLT levels between these two timepoints in the whole group. A similar relationship with increasing MLT levels emerged in RE participants, which may reflect a subtle slowing of cognitive processes in individuals who respond favorably to DS. Two interpretations are possible: (1) higher post-DS MLT levels may indicate greater sleepiness, directly impairing attention [35]; (2) a larger increase in 5-HT between PSG and DS may signal a heightened stress response or altered neurotransmission [36,37], both of which can be detrimental to cognitive precision. This supports the idea that balanced serotonergic signaling favors cognitive stability, whereas excessive stimulation—potentially due to altered SERT function—may degrade performance [36]. Neuroimaging studies demonstrate that DS disrupts prefrontal and parietal activity, regions critical for attention and executive control; dysregulated monoamine signaling within these networks likely contributes to increased error rates [38,39]. Notably, SERT expression emerged as a key modulator. In NR, an increase in SERT expression between PSG and DS was associated with fewer BEHCT errors, suggesting that efficient serotonergic reuptake may support attentional control after sleep loss. Changes in SERT expression also correlated with alterations in TMT and Stroop performance between DS and PSG timepoints, but in a task- and subgroup-specific manner. In NR, increases in SERT were correlated with longer completion times in TMT Part 1 after DS, indicating slower processing speed, whereas in RE, higher SERT expression following DS was linked to improved inhibitory control in the Stroop task, suggesting enhanced executive function. These findings suggest that SERT may modulate cognitive functions in the context of the antidepressant response to DS. Higher post-DS SERT expression in RE was associated with better executive performance and inhibitory control, indicating adaptive serotonergic regulation during DS. In contrast, in NR, altered SERT dynamics after DS were linked to slower processing speed, reflecting less efficient serotonergic modulation and a weaker cognitive and mood response to DS. These differences may arise from individual variability in circadian sensitivity, baseline serotonergic tone, or the capacity to engage compensatory neurobiological mechanisms in response to sleep loss. Overall, our findings suggest that more pronounced physiological responses to DS, such as elevated MLT levels and changes in serotonergic activity after DS, are associated with specific alterations in cognitive performance between DS and PSG timepoints, implying that individual affective responsiveness may modulate cognitive effects of DS.

Several limitations of the current study warrant consideration. First, peripheral blood measures may not accurately reflect central neurotransmission. Circulating 5-HT derives primarily from platelets and enterochromaffin cells, and although SERT can mediate limited 5-HT transport across the blood-brain barrier, this exchange is highly restricted [12,18]. Similarly, leukocyte SERT mRNA is an indirect proxy for neuronal transporter expression, though platelet SERT activity partially correlates with central measures [18]. Second, DS was short-term and performed under home-based conditions monitored by actigraphy. Variability in light exposure and nocturnal activity may have influenced MLT levels and introduced interindividual differences. Controlled laboratory protocols with fixed lighting, diet, and supervision could provide more consistent results. Moreover, actigraphy might have misclassified brief naps or motionless wakefulness, adding uncertainty. However, unlike strict laboratory protocols that minimize environmental influences, our approach preserved the participants’ natural living conditions, thereby better reflecting the clinical challenges faced by such patients. Third, the study lacked a crossover design with a repeated normal-sleep control, limiting control for circadian variation, repeated testing, and stress-related effects. Although participants were carefully monitored using sleep diaries and actigraphy to ensure protocol adherence and detailed characterization of sleep-wake patterns, which reduces causal inference regarding sleep deprivation-specific effects.

In conclusion, acute DS appears to dysregulate the TP-5-HT-MLT axis, disrupting its regulatory balance. These effects may result from metabolic redistribution of TP and related molecules, potentially involving a shift toward the kynurenine pathway. Our findings, together with existing literature, indicate that experimental DS conditions can significantly affect the functional dynamics of this axis. Moreover, the observed molecular alterations vary according to affective response following DS, suggesting distinct peripheral 5-HT and MLT profiles between individuals who do and do not experience mood improvement following sleep loss. Mood response may further contribute to cognitive changes following DS, either impairment or enhancement, through serotonergic-melatoninergic mechanisms, as reflected by the associations between BEHCT error rates and measured peripheral parameters, which may reflect cognitive vulnerability or resilience after DS.

Overall, these results emphasize the pivotal role of the gut-brain axis, whereby dietary TP intake and its peripheral metabolism may influence neuropsychiatric responses. Future research should aim to replicate clinically relevant DS paradigms, quantify kynurenine pathway metabolites, and employ experimental manipulations (e.g., precursor supplementation) to clarify directionality and causal relationships underlying these effects.

## 4. Materials and Methods

### 4.1. Protocol

The research enrolled adults between 18 and 35 years of age with a body mass index (BMI) ranging from 20 to 30 kg/m^2^. All participants provided written informed consent prior to inclusion. Individuals were excluded if they reported night or rotating shift work, pregnancy or breastfeeding, chronic illnesses, prior chemotherapy or radiotherapy, malignant neoplasms other than basal cell carcinoma, recent surgery, substance abuse, previously diagnosed sleep disorders within the past two years, ongoing infections, or intercontinental travel within the two weeks preceding enrolment.

The investigation consisted of two nights: polysomnography (PSG) and DS. Prior to PSG, participants signed consent forms and underwent a physical examination. PSG recording encompassed several channels: electroencephalography (EEG) for brain activity, electromyography (EMG) of the chin and anterior tibialis muscles to monitor muscle tone, electrooculography (EOG) for eye movement detection, a thermistor for assessment of oronasal airflow, snoring detection, body position sensors, piezoelectric belts for measurement of thoracic and abdominal respiratory effort, a unipolar electrocardiogram (ECG) for cardiac monitoring, and pulse oximetry (SpO_2_) to determine blood oxygen saturation (Alice 6, Philips-Respironics). Sleep stages were scored in 30-s epochs following the criteria of the American Academy of Sleep Medicine (AASM).

**Figure 2:**
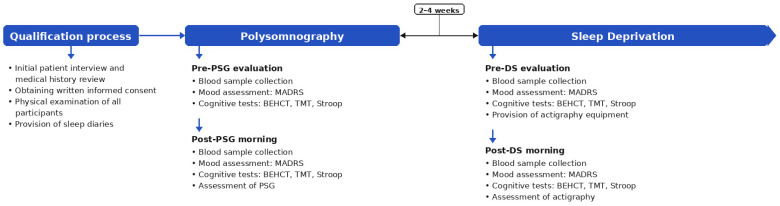
Schematic overview of the experimental design and study procedures. Abbreviations: MADRS—Montgomery-Åsberg Depression Rating Scale, BEHCT—Bimanual Eye-hand Coordination Test, TMT—Trail Making Test, Stroop—Stroop Color and Word Test, PSG—Polysomnography.

Approximately two to four weeks later, the same individuals participated in DS, which was monitored by actigraphy (GENEActiv Original, ActivInsights Ltd., UK). At both study stages (PSG and DS), venous blood samples (9 mL) and mood assessments were collected twice—in the evening before and in the morning after each session. Figure 2 illustrates the overall study design and experimental procedures.

Mood was assessed with the Montgomery-Åsberg Depression Rating Scale (MADRS), a 10-item questionnaire evaluating depressive symptoms such as sadness, tension, and sleep. Scores above seven indicate the presence of mild depression. Based on overnight changes, participants were categorized as Responders (RE), showing an improvement in MADRS score or maintaining a score < 8, or Non-Responders (NR), with no overnight improvement. The cutoff was selected based on previous studies using MADRS thresholds corresponding to minimal depressive symptom severity/remission (<8 points), as well as prior sleep deprivation studies indicating the lack of a universally established response criterion [40,41]. Cognitive performance was evaluated by the tests listed below.

**The Bimanual Eye-Hand Coordination Test (BEHCT)** is designed to assess an individual’s ability to coordinate visual input with manual movements. During the test, the participant is required to trace the outline of a star using a stylus. The stylus is manipulated via two handwheels, which control horizontal and vertical movement. The device records several performance metrics, including the total time taken to complete the task, the number of errors made (instances of crossing outside the lines), and the duration spent in incorrect positions.

**The Trail Making Test (TMT)** is a tool commonly used to evaluate executive functioning, visual-motor abilities, and cognitive processing skills. In this test, participants receive a sheet containing either scattered numbers (part A) or a combination of numbers and letters (part B). The task requires the individual to link the elements in ascending order as quickly as possible.

**The Stroop Color and Word Test** is designed to assess executive functions and the capacity to inhibit cognitive interference. During the test, participants are asked to read aloud 25 words. In the first part, the color of each word conflicts with its meaning, whereas in the second part, the color and the word meaning correspond.

The study protocol received ethical approval from the Bioethics Committee of the Medical University of Łódź (reference number: RNN/302/20/KE).

### 4.2. Biochemical and Molecular Analysis

Analyses of gene expression and serum concentrations were performed at two time points—in the morning following PSG and in the morning after DS. Serum was selected as the biological matrix for biochemical analyses in order to reduce variability associated with platelet-derived serotonin release and platelet activation. Levels of 5-HT, TP, and MLT were determined using ELISA kits (Demeditec for 5-HT, BT LAB for TP, EIAab for MLT) Demeditec Diagnostics GmbH, Kiel, Germany, and absorbance was measured at 450 nm with a BioTek 800 TS absorbance reader (Agilent Technologies, Santa Clara, CA, USA). Isolation of RNA was performed using the Trizol method (Invitrogen) and quantified with a spectrophotometer (Nanodrop Colibri, Titertek Berthold, Germany). The obtained RNA was then reverse-transcribed to cDNA (SuperScript IV First-Strand Synthesis System, Thermo Fisher Scientific, Waltham, MA, USA). Quantitative reverse transcription polymerase chain reaction (qRT-PCR) was carried out on a Rotor-Gene™ 3000 thermal cycler (Corbett Research Pty Ltd., Sydney, Australia) using a commercially available TaqMan assay kit (Thermo Fisher Scientific, Waltham, MA, USA). The reaction mixture consisted of SERT probes (Taqman), the reference gene GAPDH, nuclease-free water, a master mix reaction mixture, and cDNA. Amplification was performed for 50 cycles. Results were expressed as ΔCT and analyzed using the 2^−∆∆Ct^ method.

### 4.3. Statistical Analysis

All statistical computations were conducted using Statistica software, version 13.1PL (StatSoft, Tulsa, OK, USA). The level of statistical significance was set at *p* < 0.05. Prior to analysis, gene expression values were subjected to logarithmic transformation. The Shapiro-Wilk test was applied to assess the normality of variables distributions. Depending on the data characteristics, comparisons between groups were performed using Student’s *t*-test for normally distributed variables and Wilcoxon signed-rank test or the Mann-Whitney U test for non-normally distributed or non-parametric data. Associations between selected parameters were assessed using Spearman’s rank correlation coefficient. For each examined neurotransmitter and gene, an expression index (Δ) was determined as the ratio of post-DS to post-PSG expression values (Δ = post-DS value/post-PSG value).

## Figures and Tables

**Table 1:** Comparison of demographic, polysomnographic, molecular, and biochemical parameters between study groups.

	All (*n* = 80)	RE (*n* = 49)	NR (*n* = 31)	*p*-Value
Age	24.0 (22.0–26.0)	24.0 (23.0–27.0)	23.0 (22.0–25.0)	0.120
BMI [kg/m^2^]	22.9 ± 2.7	23.3 ± 2.6	22.3 ± 2.8	0.123
Women [*n*, %]	40 (50.0%)	21 (42.9%)	19 (61.3%)	0.169
Smoking [*n*, %]	9 (11.3%)	5 (10.2%)	4 (12.9%)	0.729
TIB [min]	536.0 (514.3–558.2)	537.0 (521.0–562.0)	530.0 (507.5–544.5)	0.091
TST [min]	407.5 (359.5–470.8)	424.0 (369.0–467.0)	396.0 (352.0–471.8)	0.486
Sleep latency [min]	40.0 (26.4–64.8)	37.5 (27.5–61.5)	41.0 (21.8–67.3)	0.933
Δ Tryptophan [µmol/L]	1.2 (0.9–1.8)	1.2 (0.8–1.8)	1.3 (1.0–2.2)	0.447
Δ Serotonin [ng/mL]	0.9 (0.7–1.1)	1.0 (0.7–1.2)	0.8 (0.7–1.1)	0.182
Δ mRNA SERT	0.6 (0.2–2.6)	0.9 (0.3–2.6)	0.5 (0.2–1.9)	0.205
Δ Melatonin [pg/mL]	1.0 (0.9–1.2)	1.0 (0.9–1.2)	1.1 (1.0–1.2)	0.323

Abbreviations: BMI—Body Mass Index, Δ—Post-DS Value/Post-PSG Value, DS—Deprivation of Sleep, NR—Nonresponders, RE—Responders, SERT—Serotonin Transporter, TIB—Time in Bed, TST—Total Sleep Time.

**Table 2:** Comparison of gene expression, hormones and amino-acid levels between two timepoints—post-PSG and post-DS.

	All			RE			NR		
	Post-PSG	Post-DS	*p*-Value	Post-PSG	Post-DS	*p*-Value	Post-PSG	Post-DS	*p*-Value
Tryptophan [µmol/L]	44.9 (30.4–68.2)	30.8 (19.9–56.9)	**0.024**	45.8 (29.7–70.2)	30.5 (20.6–62.7)	0.165	44.6 (33.4–64.6)	31.2 (17.9–40.9)	0.055
Serotonin [ng/mL]	80.0 (49.0–120.0)	84.3 (58.8–119.0)	0.120	81.8 (58.0–135.2)	85.0 (59.6–116.0)	0.701	63.8 (30.7–86.7)	76.6 (53.8–120.3)	**0.043**
mRNA SERT	−3.8 (−4.1–−3.4)	−3.6 (−3.8–−3.3)	0.286	−3.6 (−3.9–−3.3)	−3.6 (−3.8–−3.3)	0.794	−4.0 ± 0.6	−3.5 ± 0.7	0.088
Melatonin [pg/mL]	457.0 (391.7–511.2)	423.6 (392.7–464.2)	**0.043**	451.3 (392.8–513.1)	431.3 (389.4–487.0)	0.369	459.3 ± 73.2	423.6 ± 57.1	**0.024**

Abbreviations: NR—Nonresponders, RE—Responders, SERT—Serotonin Transporter, PSG—Polysomnography, DS—Deprivation of Sleep. Data are presented as median (IQR) or mean ± SD. Bolded text indicates statistical significance.

**Table 3:** Correlations between cognitive changes and molecular/biochemical parameters between post-PSG and post-DS mornings.

	Δ Tryptophan			Δ Serotonin			Δ mRNA SERT			Δ Melatonin		
	All	RE	NR	All	RE	NR	All	RE	NR	All	RE	NR
Δ BEHCT number of errors	R = −0.21, *p* = 0.072	**R = −0.32, *p* = 0.029**	R = −0.03, *p* = 0.897	**R = 0.25, *p* = 0.028**	R = 0.25, *p* = 0.089	R = 0.22, *p* = 0.250	R = −0.17, *p* = 0.250	R = −0.08, *p* = 0.684	**R = −0.54, *p* = 0.032**	**R = 0.28, *p* = 0.018**	**R = 0.41, *p* = 0.005**	R = −0.05, *p* = 0.805
Δ Stroop test part 1	R = 0.07, *p* = 0.540	R = 0.01, *p* = 0.955	R = 0.13, *p* = 0.478	R = 0.02, *p* = 0.850	R = 0.11, *p* = 0.465	R = −0.07, *p* = 0.711	R = −0.20, *p* = 0.188	R = −0.26, *p* = 0.069	R = 0.06, *p* = 0.762	R = 0.03, *p* = 0.776	R = −0.02, *p* = 0.900	R = 0.03, *p* = 0.883
Δ Stroop test part 2	R = 0.05, *p* = 0.661	R = 0.02, *p* = 0.885	R = 0.10, *p* = 0.600	R = 0.14, *p* = 0.220	R = 0.16, *p* = 0.273	R = 0.09, *p* = 0.644	R = −0.25, *p* = 0.078	**R = −0.29, *p* = 0.039**	R = 0.04, *p* = 0.828	R = 0.03, *p* = 0.813	R = −0.03, *p* = 0.857	R = 0.12, *p* = 0.516
Δ TMT part 1	R = 0.08, *p* = 0.500	R = 0.14, *p* = 0.336	R = 0.07, *p* = 0.723	R = 0.12, *p* = 0.296	R = 0.19, *p* = 0.186	R = 0.10, *p* = 0.583	R = 0.17, *p* = 0.271	R = 0.07, *p* = 0.642	**R = 0.37, *p* = 0.045**	R = 0.14, *p* = 0.225	R = 0.13, *p* = 0.382	R = 0.15, *p* = 0.453
Δ TMT part 2	R = 0.02, *p* = 0.880	R = −0.08, *p* = 0.588	R = 0.25, *p* = 0.178	R = 0.10, *p* = 0.404	R = 0.05, *p* = 0.727	R = 0.22, *p* = 0.234	**R = 0.28, *p* = 0.014**	**R = 0.38, *p* = 0.007**	R = 0.18, *p* = 0.349	R = 0.18, *p* = 0.110	R = 0.22, *p* = 0.130	R = −0.02, *p* = 0.918

Abbreviations: NR—Nonresponders, RE—Responders, SERT—Serotonin Transporter, BEHCT—Bimanual Eye-hand Coordination Test, TMT—Trail Making Test, Δ—Post-DS Value/Post-PSG Value. Bolded text indicates statistical significance.

## Data Availability

Data available on request from the authors.

## Abbreviations

The following abbreviations are used in this manuscript:

DS	Deprivation of Sleep
TP	Tryptophan
5-HT	5-Hydroxytryptamine (Serotonin)
SERT	Serotonin Transporter
MLT	Melatonin
PSG	Polysomnography
MADRS	Montgomery-Åsberg Depression Rating Scale
BEHCT	Bimanual Eye-Hand Coordination Test
TMT	Trail Making Test
BMI	Body Mass Index
EEG	Electroencephalography
EMG	Electromyography
EOG	Electrooculography
ECG	Electrocardiogram
RE	Responders
NR	Non-Responders
GAPDH	Glyceraldehyde-3-Phosphate Dehydrogenase
qRT-PCR	Quantitative Reverse Transcription Polymerase Chain Reaction
IDO	Indoleamine 2,3-Dioxygenase
